# Distribution of the transposable elements *bilbo *and *gypsy *in original and colonizing populations of *Drosophila subobscura*

**DOI:** 10.1186/1471-2148-8-234

**Published:** 2008-08-14

**Authors:** María Pilar García Guerreiro, Blanca E Chávez-Sandoval, Joan Balanyà, Lluís Serra, Antonio Fontdevila

**Affiliations:** 1 Grup de Biología Evolutiva. Departament de Genètica i Microbiologia, Facultat de Biociències, Universitat Autònoma de Barcelona, 08193 Bellaterra (Barcelona), Spain.; 2 Grup de Biología Evolutiva. Department de Genètica, Facultat de Biologia, Universitat de Barcelona, 08071 Barcelona, Spain.

## Abstract

**Background:**

Transposable elements (TEs) constitute a substantial amount of all eukaryotic genomes. They induce an important proportion of deleterious mutations by insertion into genes or gene regulatory regions. However, their mutational capabilities are not always adverse but can contribute to the genetic diversity and evolution of organisms. Knowledge of their distribution and activity in the genomes of populations under different environmental and demographic regimes, is important to understand their role in species evolution. In this work we study the chromosomal distribution of two TEs, *gypsy *and *bilbo*, in original and colonizing populations of *Drosophila subobscura *to reveal the putative effect of colonization on their insertion profile.

**Results:**

Chromosomal frequency distribution of two TEs in one original and three colonizing populations of *D. subobscura*, is different. Whereas the original population shows a low insertion frequency in most TE sites, colonizing populations have a mixture of high (frequency ≥ 10%) and low insertion sites for both TEs. Most highly occupied sites are coincident among colonizing populations and some of them are correlated to chromosomal arrangements. Comparisons of TE copy number between the X chromosome and autosomes show that *gypsy *occupancy seems to be controlled by negative selection, but *bilbo *one does not.

**Conclusion:**

These results are in accordance that TEs in *Drosophila subobscura *colonizing populations are submitted to a founder effect followed by genetic drift as a consequence of colonization. This would explain the high insertion frequencies of *bilbo *and *gypsy *in coincident sites of colonizing populations. High occupancy sites would represent insertion events prior to colonization. Sites of low frequency would be insertions that occurred after colonization and/or copies from the original population whose frequency is decreasing in colonizing populations. This work is a pioneer attempt to explain the chromosomal distribution of TEs in a colonizing species with high inversion polymorphism to reveal the putative effect of arrangements in TE insertion profiles. In general no associations between arrangements and TE have been found, except in a few cases where the association is very strong. Alternatively, founder drift effects, seem to play a leading role in TE genome distribution in colonizing populations.

## Background

TEs are widely distributed in eukaryotes, representing 50% of the human genome [1], 15% of the Drosophila genome, and up to 70% in *Zea mays *[2]. Because of their capacity of transposition they are able to invade the genome and promote insertional mutations and chromosomal rearrangements. Recurrent mobility allows them to persist in spite of their harmful effects in the host [3]. Most of the proposed models in population dynamic studies [4-8] suggest that TEs are able to invade the genome if their transposition rate is enough to balance out opposing forces as excision and selection against deleterious insertions and chromosomal arrangements. Yet, these models, often too general, do not consider that each element behaves depending on both its own characteristics and the history of the population to which it belongs. This challenge to standard reasoning is most relevant in colonizing populations [9]. Several authors have suggested that bursts of transposition could be induced in colonization by the foreign, often stressful, environment faced by the founders of colonizing populations [10,11]. Moreover, colonizing populations are subjected to well documented founder, drift effects [12]. Both processes generate population instabilities that may incorporate new variables to the interpretation of TE occupancy profiles in colonizing populations. These considerations qualify the study of TEs in colonization as of prime interest to understanding their invasive dynamics and putative evolutionary role in populations.

Colonization effects on TEs were studied in Drosophila species [9,11,13] showing that this process plays an important role in the TE chromosomal distribution. In particular studies in colonizing populations of *D. buzzatii *showed a TE bimodal distribution with sites either highly occupied, in a few cases, or showing low insertion occupancy, in most cases. Molecular studies of TE copies from high and low occupied sites [14] strongly indicated that the most reliable explanation of the observed bimodal distribution is that a founder effect followed by genetic drift occurred during the colonization process. These results notwithstanding, valid for *D. buzzatii*, cannot be generalized to other colonizing Drosophila species, with different genomic characteristics, and subjected to different environmental pressures.

*D. subobscura*, a Paleartic species belonging to the obscura group [15] and characterized by a rich inversion polymorphism [16], has colonized North and South America almost 30 years ago [17,18]. It was found for the first time in Puerto Montt (Chile) in 1978 [19] and later near Port Townsend in Washington (USA) in 1982 [20]. Thereafter this species showed a rapid spread and adaptation to the new colonized environment in form of latitudinal clines for chromosomal polymorphism and body size that paralleled the Paleartic clines [17,18,21]. Main after-colonization population effects were the presence of allelic lethal genes in different populations [22], the low genetic variability of mtDNA [23,24] and the reduction of microsatellite allele numbers [25] compared to original founder populations. These are expected outcomes of the founder drift effect of colonization. However nothing is known of the impact of colonization on the TE chromosomal distribution in this species.

Here we present the study of the distribution of two TEs, *gypsy *and *bilbo*, in original and colonizing populations of *D. subobscura*. Results show that TE frequency distribution differ between original and colonizing populations in a way that colonization, chromosomal inversion polymorphism and particular characteristics inherent to each element can provide a sufficient likely explanation. In this paper we particularly emphasize the importance of population structure and history to explain TE distribution in natural populations.

## Results

### Chromosomal distribution of *bilbo *and *gypsy*

**Figure 1:**
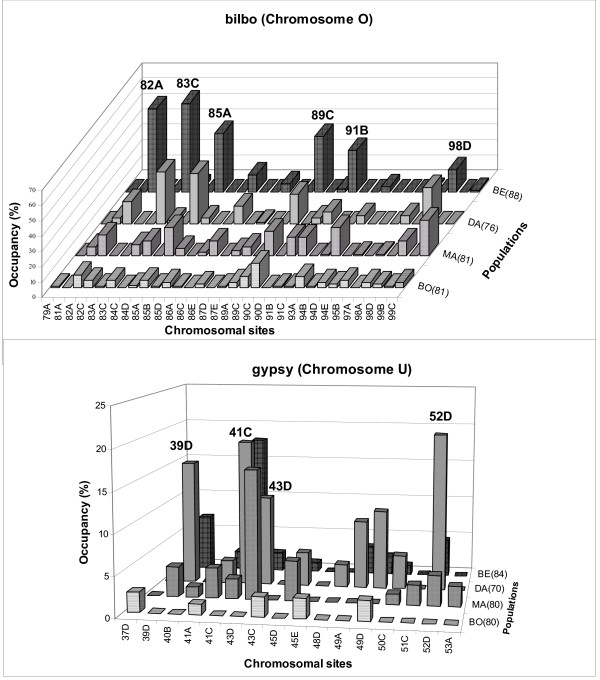
**Distribution of bilbo and gypsy in chromosomes O and U, respectively, from colonizing (DA: Davis, BE: Bellingham, MA: Maipú) and original populations (BO: Bordils) of D. subobscura**. Number of haploid genomes analyzed are given in parenthesis.

We analyzed the distribution of *bilbo *and *gypsy *in polytene chromosomes of *D. subobscura*. Fig. 1 shows two examples of chromosomal distribution: *bilbo *in chromosome O and *gypsy *in chromosome U. A different distribution pattern is observed, in general, when we compare colonizing and original populations. Colonizing populations present insertion frequencies of *bilbo *and *gypsy *higher than those of the original population. In general the same distribution pattern is observed for the rest of chromosomes. Ten sites (7A, 16A, 20A, 45C, 58D, 74D 82A, 83C, 85A, 89C) show a *bilbo *insertion polymorphism greater than 32% in at least one colonizing population. *Gypsy *insertion frequencies are lower than those of *bilbo *with an occupancy of more than 10% in eight chromosomal sites (39D, 41C, 43D, 49D, 52D, 63C, 71B, 74D). Differences in occupancy profiles between original and colonizing populations are represented in Table 1 that shows the distribution of the number of times that each site is occupied in the studied sample. Thus, in *bilbo *the occupancy frequency ranges from 1 to 51 times in colonizing populations and only from 1 to 19 in the original population. Although *gypsy *shows a low occupancy profile compared to *bilbo *(colonizing populations range: 1–15; original population range: 1–5), the occupancy rate of both TEs in colonizing populations is greater than in the original population. The highest *bilbo *insertion frequencies are observed, in decreasing order, in Bellingham, Maipú and Davis. In the original population of Bordils, the highest insertion frequency corresponds to one site observed 16 times.

**Table 1:** Occupancy profiles of euchromatic sites in original and colonizing populations

TE	Populations	Occupancy profiles																	
			**1**	**2**	**3**	**4**	**5**	**6**	**7**	**8**	**9**	**10**	**11**	**12**	**13**	**14**	**15**	**16**	**17–51**

**bilbo**	**Colonizing**	DA	7	6	9	4	1	7	0	2	2	1	5	1	1	0	1	0	7
		BE	3	5	3	2	4	2	1	2	1	2	0	0	4	1	2	2	10
		MA	8	8	4	7	4	2	2	5	2	3	2	3	2	1	2	1	12
	**Original**	BO	32	19	21	11	6	2	5	1	2	0	1	0	1	0	3	1	1

**gypsy**	**Colonizing**	DA	8	4	4	0	1	2	2	2	0	0	1	0	1	1	0		
		BE	11	5	1	2	2	2	1	0	0	0	0	0	0	0	1		
		MA	9	10	6	3	2	1	1	0	0	0	0	0	2	0	0		
	**Original**	BO	10	12	5	2	1	0	0	0	0	0	0	0	0	0	0		

Population origin: Davis (DA), Bellingham (BE), Maipú (MA), Bordils (BO).

Occupancy profile: number of times that each site is occupied in populations.

Table 2 lists the means and variances of copy number for *bilbo *and *gypsy *per chromosome and haploid genome. The mean copy number of both TEs for the whole genome (HG) is always higher in colonizer populations than in the original one. The *bilbo *mean copy number differs greatly among chromosomes ranging from 2.58 copies in chromosome O from Bellingham to 0.55 in chromosome J from Davis. In fact chromosome J hosts the lowest number of *bilbo *in all populations. A different scenario is found for *gypsy *in which the A (X) chromosome contains the lowest number of insertions, in colonizer and original populations alike. However, among the autosomes J is the least occupied in all populations. Deviation from Poisson distribution was tested by chi-square goodness of fit tests (for details, see additional files 1 and 2) pooling adjacent classes with low expected numbers. In colonizing populations *bilbo *distribution in each chromosome fits a Poisson distribution. *Gypsy *deviates from a Poisson distribution in E chromosome from Davis and Bellingham and in U chromosome from Maipú. When the whole genome is considered both TEs follow a Poisson distribution in the original population and deviate in all colonizing ones, except for *bilbo *in Bellingham and gypsy in Maipú. For this element the general trend in colonizing populations is a lower than expected number of genomes with a single copy and an excess of genomes with three or more copies (see Table 1). An alternative test was performed using dispersion coefficients (DC), which measure the ratio between the variance (V_n_) and the mean (m) (DC = V_n_/m, see table 2). DC of 1 indicates that TE distribution is Poisson, and DC > 1 or DC < 1 indicates contagious or repulsive distributions, respectively. When the haploid genome is considered, there is a general tendency towards DCs > 1 for both elements in all populations except for *gypsy *in Maipú (these results are due to the greater effect of some chromosomes in the final result of the test).

**Table 2:** Tests of the Poisson distribution of *bilbo *and *gypsy *per chromosome and haploid genome.

		Populations									
		
		DA (76)					BE (88)				
		
TE	Ch.	m	V_n_	DC	**χ**^2^	df	m	V_n_	DC	**χ**^2^	df
	**A**	0.84	0.72	0.86	0.61	2	1.07	0.77	0.73	2.68	2
	**J**	0.55	0.49	0.89	0.11	1	0.76	0.67	0.88	1.61	2
	**U**	1.01	0.87	0.86	5.74	3	1.11	1.00	0.90	1.46	3
	**E**	1.38	1.73	1.25	4.70	4	0.82	0.84	1.03	0.69	3
	**O**	1.71	1.67	0.98	3.47	5	2.58	1.80	0.70	7.06	4
**bilbo**	**HG**	5.50	9.40	1.71**	32.56**	11	6.34	7.79	1.23	18.61	11
		
		**MA (81)**					**BO (81)**				
		
		**m**	**V_n_**	**DC**	**χ**^2^	**df**	**m**	**V_n_**	**DC**	**χ**^2^	**df**
		
	**A**	1.75	1.69	0.96	1.54	5	1.06	0.71	0.67	8.11	2
	**J**	0.91	0.93	1.02	0.25	3	0.75	0.81	1.08	13.65*	4
	**U**	1.54	1.28	0.83	4.15	4	0.84	0.69	0.82	1.14	2
	**E**	1.80	1.66	0.92	1.70	4	1.09	1.70	1.57**	29.79**	5
	**O**	1.79	1.99	1.11	6.53	5	0.96	1.28	1.34	9.58	3
	**HG**	7.80	19.51	2.50**	254.34**	18	4.70	5.76	1.22	11.88	10

		**DA (70)**					**BE (84)**				
		
		**m**	**V_n_**	**DC**	**χ**^2^	**df**	**m**	**V_n_**	**DC**	**χ**^2^	**df**
		
	**A**	0	0	-	-	-	0.02	0.02	1.00	-	-
	**J**	0	0	-	-	-	0.02	0.02	1.00	-	-
	**U**	0.98	1.26	1.28	5.53	3	0.43	0.49	1.14	1.76	3
	**E**	0.58	0.97	1.66**	44.10**	4	0.33	0.56	1.69**	38.10	3
	**O**	0.04	0.04	0.97	-	-	0.09	0.11	1.17	1.38	1
**gypsy**	**HG**	1.61	2.44	1.51*	14.89	5	0.90	1.60	1.77**	33.18**	4
		
		**MA (80)**					**BO (80)**				
		
		**m**	**V_n_**	**DC**	**χ**^2^	**df**	**m**	**V_n_**	**DC**	**χ**^2^	**df**
		
	**A**	0.12	0.16	1.29	4.46	1	0.02	0.02	1.00	-	-
	**J**	0.14	0.12	0.87	-	-	0.10	0.12	1.16	1.26	1
	**U**	0.42	0.27	0.64	8.49*	1	0.11	0.10	0.90	-	-
	**E**	0.39	0.32	0.82	1.43	1	0.30	0.34	1.13	2.27	1
	**O**	0.27	0.28	1.01	0.12	1	0.24	0.23	0.99	0.00	1
	**HG**	1.35	1.27	0.94	11.48	4	0.77	0.88	1.14	3.44	2

TE: Transposable elements; Ch: chromosome; HG: haploid genome; m: mean copy number; V_n_: variance of copy number; Numbers of haploid genomes are in parenthesis; DC: dispersion coefficient (V_n_/m); *P < 0.05; **P < 0.01. Bonferroni's correction was applied. See Table 1 for population origin

**Figure 2:**
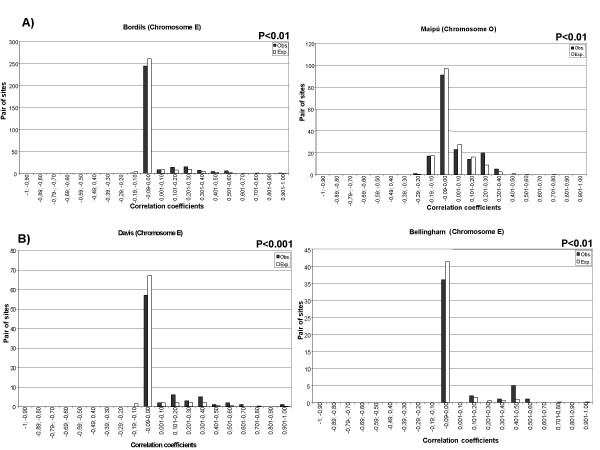
**Observed and expected frequency distributions of correlation coefficients between all pairs of sites in natural populations: A) ***bilbo *in chromosome E from Bordils and O from Maipú. **B)***gypsy *in chromosome E from Davis and Bellingham.

Because in some cases TE sites seem to be distributed in a contagious way (DC > 1), linkage disequilibrium was computed for each pair of sites by way of 2 × 2 contingency tables [26]. Linkage disequilibrium between TE sites could be responsible of the non-random distribution detected in some cases. The observed distribution of correlation coefficients between all paired sites was compared to the expected distribution in absence of linkage disequilibrium using Fisher's hypergeometric formula [27]. Figure 2 depicts, as an example, the correlation coefficient distributions (pooled in intervals of 0.1) of *bilbo *in chromome E from Bordils and in chromosome O from Maipú; and of *gypsy *in chromosome E from Davis and Bellingham. Tests were significant in most cases where a deviation from Poisson distribution was observed. Moreover we also found significant results in some cases where departures from Poisson distribution were not detected (e.g *bilbo *on chromosome O of Maipú). The general trend is a defect of class (-0.09–0.00) and an excess of some positive correlation classes. This indicates that some sites tend to stay together, as indicated by a DC > 1. This tendency was observed in all cases where deviations from Poisson distribution were observed, except for *gypsy *in chromosome U from Maipú where there is an overabundance in class (-0.09–0.00) and the DC is lower than 1.

### Copy number comparisons among chromosomes

Montgomery et al [28] proposed that selection against TE insertions would lead to a lower number of TE copies in chromosome X than in autosomes due to the stronger deleterious effect of recessive insertional mutations in the X chromosome of hemizygous males. In order to test this hypothesis we compared the copy number in the A (X) chromosome with that in autosomes. To estimate the expected number of insertions we multiply the relative proportion of chromatin of each chromosome by the number of total insertions in the population. The relative proportion of chromatin is that reported by Stumm-Zollinger and Goldschmidt [29] corrected by eliminating the dot chromosome, not included in our analyses. If TEs are randomly distributed, we expect a TE copy number per chromosome proportional to the amount of chromatin.

Observed and expected proportions were compared by a G test [30] among all chromosomes (G_a_), between the A (X) chromosome and autosomes (G_b_), and among autosomes (G_c_), as indicated in Table 3. G_b _values were significant for *gypsy *in all populations, and for *bilbo *only in Maipú and Bordils. Because some differences may be due to high insertion sites, additional analyses were done after eliminating these sites. After elimination the significance was maintained for *gypsy *in all populations except in Maipú, and removed for *bilbo*. In general *gypsy *shows a low copy number in the A (X) chromosome compared to autosomes. However, this is not the rule for *bilbo *where Maipú and Bordils show a high copy number in A (X). Interestingly, those populations that display *gypsy *copy number differences between A (X) and autosomes, show also significant differences among autosomes (G_c_), specially in colonizer populations where chromosomes E and O show a higher copy number than expected.

**Table 3:** Comparison of the proportion of *gypsy *and *bilbo *sites among chromosomes, autosomes and between chromosome A and autosomes

TE		gypsy				bilbo			
	
Ch.	P. chromat	DA	BE	MA	BO	DA	BE	MA	BO
**A(X)**	0.16	0.00	0.03	0.09	0.03	0.15	0.17	0.22	0.23
**J**	0.20	0.01	0.03	0.10	0.13	0.10	0.12	0.12	0.16
**U**	0.19	0.61	0.48	0.31	0.14	0.18	0.17	0.20	0.18
**E**	0.20	0.36	0.37	0.29	0.39	0.25	0.13	0.23	0.23
**O**	0.25	0.03	0.10	0.20	0.31	0.31	0.40	0.23	0.20

	**Df**								
**Ga**	4	197.23** (115.82**)	71.18** (61.31**)	22.26** (5.08)	21.40** (21.40**)	36.98** (0.08)	87.16** (16.98**)	41.82** (4.80)	15.67 (8.66)
**Gb**	1	40.48** (26.15**)	15.24** (17.20**)	4.63* (1.14)	11.06**	0.36 (4.8)	0.08 (3.47)	15.56** (2.9)	9.69** (0.52)
**Gc**	3	156.75** (89.66**)	55.93** (44.11**)	17.63** 3.94	10.34*	36.61** (3.47)	87.08** (13.50*)	26.26** (1.87)	5.97 (8.10*)

**Ga**									
Total	12	290.67**(182.21**)				165.96**(30.06**)			
Pooled	4	230.15*(110.30**)				102.01**(1.8)			
H	8	60.52**(71.90**)				63.95**(28.23**)			
**Gb**									
Total	3	60.36**(44.50**)				16.00**(11.20**)			
Pooled	1	44.97**(23.78**)				5.77*(1.09)			
H	2	15.38**(20.71**)				10.24*(10.10*)			
**Gc**									
Total	9	230.32**(137.71**)				149.95**(18.85)			
Pooled	3	185.18**(86.52**)				96.25**(0.72)			
H	6	45.13**(51.19**)				53.71**(18.12*)			

P. chromat: Proportion of chromatin. Ga: Comparison of the proportion of TEs among chromosomes. Gb: Comparison of the proportion of TEs between chromosome A (X) and autosomes. Gc: Comparison of the proportion of TEs among autosomes; H: Heterogeneity test between colonizing populations; Df: Degrees of freedom; Pooled: Only colonizing populations; *P < 0.05; **P < 0.001. Bonferroni's correction was applied. Test values excluding high insertion frequency sites are in parenthesis.

In general, copy number tend to be higher for *bilbo *in chromosome O and for *gypsy *in chromosome U in all colonizing populations, (except *bilbo *in Maipú), whereas in the original population the E chromosome hosts the highest proportion of *gypsy *and *bilbo*. In order to determine if chromosomal differences have the same tendency in colonizing populations, heterogeneity (H) tests were performed for comparisons among chromosomes, between A (X) and autosomes and among autosomes. Table 3 shows that all cases were heterogeneous for both TEs. However when Maipú is excluded from the analyses and high insertion frequency sites are eliminated, Bellingham and Davis become homogeneous for *bilbo *(data not shown).

### Correlation studies between high frequency sites and chromosomal arrangements

All five pairs of acrocentric chromosomes of *D. subobscura *are polymorphic for inversions. Frequencies of chromosomal arrangements show clinal variation correlated with latitude in Paleartic populations [31,32] and clines that follow the same latitudinal gradient evolved in recent colonizing populations in both hemispheres of the Americas [17,18]. These parallel observations across continents provided a natural experiment that supports the adaptative role of the chromosomal inversion polymorphism.

Frequencies of chromosomal arrangements in the analyzed populations of this work are summarized in Table 4. Each arrangement is conventionally designed by the letter of the chromosome in which it occurs, followed by a combination of digits that identify the set of inversions included in it [16]. Arrangement frequencies are of the same order of magnitude as those previously reported, including the North-South latitudinal variation of most arrangements [21,31,32]. However it is interesting to note that Maipú presents a higher O_St _frequency than expected according to its latitude.

**Table 4:** Frequencies of chromosomal arrangements in natural populations

Arrangements	Populations			
	
	DA	BE	MA	BO
**Ast**	0.57	0.67	0.55	0.53
**A1**	-	-	-	0.17
**A2**	0.43	0.33	0.45	0.30

**Jst**	0.41	0.45	0.26	0.40
**J1**	0.59	0.55	0.74	0.60

**Ust**	0.32	0.42	0.50	0.17
**U1**	-	-	-	0.03
**U1+2**	0.39	0.40	0.20	0.72
**U1+2+8**	0.29	0.18	0.30	0.07
**U1+2+3**	-	-	-	0.01

**Est**	0.54	0.74	0.59	0.53
**E8**	-	-	-	0.01
**E1+2**	0.07	0.05	-	0.30
**E1+2+9**	0.12	0.01	0.08	0.03
**E1+2+9+12**	0.05	0.15	0.23	0.12
**E1+2+9+3**	0.22	0.05	0.10	0.01

**Ost**	0.08	0.23	0.22	0.22
**O2**	-	-	-	0.01
**O5**	0.01	0.14	-	-
**O7**	-	-	0.01	0.04
**O3+4**	0.20	0.11	0.30	0.32
**O3+4+7**	0.17	0.01	0.21	0.04
**O3+4+2**	0.34	0.19	0.22	0.06
**O3+4+8**	0.20	0.32	0.04	0.30
**O3+4+23+2**	-	-	-	0.01

-: Arrangement absent

Some authors consider recombination as the main factor determining the chromosomal distribution of TEs [33,34], but see [34]. The model of ectopic exchange, predicts a negative correlation between recombination rate and TE copy number if ectopic exchange is reduced in parallel with regular meiotic recombination rate [35,36]. Under this model, TEs are expected to be more abundant in regions of low recombination as inversions or inversion break-points. In these regions the probability of induction of deleterious rearrangements produced by unequal recombination between TEs, is low because most of the time inversions will be found in heterozygous state (recombination is suppressed inside). Experimental evidences [37-39] suggest that TEs are responsible of chromosomal inversions in natural populations of Diptera and are particularly abundant inside and near inversion break-points [6,7,40].

In order to know whether an association between high insertion sites and arrangements exist, we computed the product-moment correlation coefficient (r) for high-frequency sites (Table 5). We observed two *bilbo *sites of particular interest (67A and 89C) that show the highest correlation coefficients. The 67A site is located inside the breakpoint of arrangement E_12 _and is significantly associated with E_1+2+9+12 _in Davis (r = 0.64) and Maipú (r = 0.85). The 89C site is located near the break-point of O_8 _arrangement and is significantly correlated with arrangement O_3+4+8 _in Davis (r = 0.58) and Bellingham (r = 0.34), and only marginally (r = 0.26) in Maipú. Other instances of significant associations are not so easily explained because sites are external to inversion breakpoints. Thus, highly occupied 74D *bilbo *site is located outside of chromosomal inversions, yet, it is also significantly associated with E_1+2+9+12 _in Davis (r = 0,33) and Maipú (r = 0,24). This site is also highly occupied by *gypsy *but in this case associations are not significant. In other cases we observe associations of sites inside highly frequent inversions where the crossing-over is not reduced. This is the case of 11B *bilbo *site, for example, negatively associated to A_2 _arrangement in all populations except Bellingham but located inside it.

**Table 5:** Correlation coefficients between chromosomal arrangements and high insertion frequency (HF) sites

		Populations							
		
		DA		BE		MA		BO	
**HF sites of bilbo**	**Arrang.**	**r**	**q-value**	**r**	**q-value**	**r**	**q-value**	**r**	**q-value**

11B	A2	-0.13	(0.42)	0.35**	(10^-7^)	0.28*	0.02	-0.07	(0.99)
20A	J1	0.24	(0.11)	-	-	-0.06	(0.39)	-0.04	(0.99)
43B	Ust	-0.08	(0.46)	0.04	0.44	0.23*	(0.04)	0.24	(0.99)
45C	Ust	0.11	(0.56)	0.20	(0.08)	0.40**	(3.10^-7^)	0.05	(0.99)
	U1+2	-0.13	(0.59)	0.22*	(0.04)	-0.19	0.08	0.02	(0.99)
45D	Ust	0.15	(0.42)	0.49**	(3.10^-7^)	--	--	--	--
	U1+2	-0.02	(0.86)	-0.34**	(2.10^-3^)	--	--	--	--
53A	Ust	-0.20	(0.15)	-0.35**	(3.10^-7^)	-0.22*	(0.04)	-0.05	(0.99)
	U1+2	0.26	(0.11)	0.05	0.44	0.18	(0.08)	0.07	(0.99)

57D	E1+2+9	-	-	0.25	(0.12)	-0.10	(0.44)	0.57	(0.41)
	E1+2+9+3	-0.06	(0.72)	0.17	(0.15)	0.28*	(0.04)	-0.02	(0.99)
59C	Est	0.36**	(5.10^-3^)	-	-	-	-	-0.02	(0.80)
67A	Est	-0.15	(0.42)	-0.25*	(0.04)	-0.69**	(4.10^-8^)	-0.03	(0.99)
	E1+2+9+12	0.64**	(1.10^-4^)	-	-	0.85**	(4.10^-8^)	0.22	(0.51)
74D	E1+2+9+12	0.33*	(0.04)	-	-	0.24*	(0.04)	0.13	(0.99)

82A	Ost	0.12	(0.63)	-0.10	(0.28)	0.29*	(0.02)	-0.16	(0.99)
83C	O3+4+7	0.00	(0.72)	0.09	(0.08)	-0.14	(0.17)	0.56	(0.11)
85A	O3+4	-0.14	(0.46)	0.27*	(0.02)	0.10	(0.17)	0.03	(0.99)
	O3+4+7	-0.17	(0.41)	0.08	0.53	-0.24*	(0.04)	-0.04	(0.99)
89C	O3+4	-0.16	0.42	-0.25*	(0.03)	-0.21	(0.06)	-0.09	(0.99)
	O3+4+2	-0.36**	(5.10^-3^)	-0.31**	(7.10^-3^)	0.09	(0.22)	0.07	(0.99)
	O3+4+8	0.58**	(1.10^-4^)	0.34**	(4.10^-3^)	0.26	(0.05)	0.02	(0.99)
91B	Ost	0.28	(0.16)	0.02	(0.12)	0.32*	(0.03)	-0.06	(0.99)
	O5	-0.03	(0.86)	0.28*	(0.02)	--	--	--	--
98D	O3+4+2	0.05	(0.72	0.12	(0.18)	0.32*	(0.03)	0.29	(0.66)

**HF sites of gypsy**									
41C	U1+2	0.35*		0.52**		0.25*		--	
52D	U1+2	0.32*		0.28*		-0.12		--	
63C	Est	--		--		-0.46**		--	
	E1+2+9+12	--		--		0.45**		--	
74D	E1+2+9+3	0.31		-0.09		0.34		-0.04	

Only high insertion sites showing correlation coefficient values either significant or higher than 0.20 at least in one population, are considered. Arrang: arrangement; r: Correlation coefficient; -: indicates cases where correlations cannot be computed because of low ETs copy number; --: indicates the lack of a site or an inversion in the population). See Table 1 for population origin. *P < 0.05; **P < 0.01. Q-value and Bonferroni corrections were applied to *bilbo *and *gypsy *respectively

## Discussion

### *Bilbo *and *gypsy *distributions are different in original and colonizer populations

Results show a clear differential TE distribution in original and colonizing populations. While in the original population most sites have low insertion frequencies, colonizing populations present some highly occupied sites, with frequencies higher than 50% for *bilbo *and close to 20% for *gypsy*. Interestingly, most of them are common to all populations. Mean copy number of both elements is higher in colonizing populations than in the original one due to the presence of these highly occupied sites.

Low occupied sites would represent insertions occurred after colonization and/or copies from the original population whose frequency is decreasing in colonizing populations. An argument in favour of the former hypothesis is the existence of unique sites that would correspond to new transpositions (i.e. site 48D of gypsy), while the latter hypothesis explains the existence of low-occupancy original sites common to different populations (i.e. site 41A of gypsy or 85B of bilbo).

High insertion frequency sites are most likely due to a founder event during the colonization process (the founder hypothesis), as previously reported in other Drosophila species [9,11,13]. In *D. buzzatii *this hypothesis was also verified by molecular studies showing identical *Osvaldo *retrotransposon structures and flanking genomic sequences in high insertion frequency sites from different colonizing populations [14].

In this study two lines of evidence support the founder hypothesis. First, the two studied TEs belong to different subclasses, yet they show a similar population behaviour. Second, most highly occupied sites are located in colonizing population chromosomes, although some exceptions occur for *bilbo *whose insertion frequency exceeds 10% in 9 sites in the original population of Bordils. All these sites correspond also to high insertion sites in colonizing populations, except 90C site and 21A, which are, respectively, free of insertions or occupied at low frequency in America.

The presence of high frequency sites in the original population could be a consequence of the transposition mechanism of *bilbo*, a LINE element. It has been shown that LINE elements (L1) make 5' truncated copies during their transposition mechanism indicating that 5' sequences are not absolutely necessary to insertion [41-43]. In fact, the majority of the L1 copies present in mammalian genomes are 5' truncated with a length of not more than 1 kb [1,44]. We can think that selection against truncated, "dead-on-arrival" (DOA) copies should be weak because they are not transcribed, potentially immobile and shorter than full copies. Thus, deleted copies could persist in some genomic regions without being completely eliminated by natural selection. In fact, some Drosophila TE families (most of them LINE like elements) seem to be only marginally affected by purifying selection, reaching high insertion frequencies in euchromatin [45].

On the other hand, some of *bilbo *high frequency sites from Bordils could be explained by the dragging effect from the rich inversion polymorphism of *D. subobscura*. For example the 67A site located in the break-point of E_12 _arrangement presents highly significant correlations with this arrangement in 2 out of 3 colonizing populations. In Bordils, this correlation is not significant because of the lower frequency of this arrangement in this population. Arrangements of chromosome E cover approximately 75% of its length and it is not rare to find this kind of associations. In this chromosome another high insertion frequency site (74D) shows association with the same E_1+2+9+12 _chromosomal arrangement. This site corresponds to a heterochromatic telomeric site where it is not rare to find an accumulation of TE insertions. In fact *gypsy *is inserted also in this chromosomal site at occupation rates that range from 1.3 to 11.4%. Accumulation of TEs in heterochromatin is well documented in *D. melanogaster *where a significant excess of insertions were reported in heterochromatin, dot and Y chromosomes alike [46-49].

Seasonal fluctuations in population frequency of chromosomal rearrangements can modify recombination rates and associations between arrangements and genes. In *D. subobscura *no seasonal fluctuations were reported in some works [50,51], but fluctuations and seasonal changes of associations between chromosomal inversions and allozymes were reported in others, specially in the O chromosome from original populations [52,53]. In the present case, we observe no associations between insertions and specific chromosomal arrangements in the original population, but we do detect this kind of associations in colonizing populations (where fluctuations were not studied). However, changes in associations between chromosomal arrangements and chromosomal sites do not follow a definite trend. As an example, the U_ST _arrangement, whose frequency has increased in all colonizing populations, shows a positive association to 43B and 45C sites but a negative one to 53A site in Maipu. This is a rather odd outcome since increase of rearrangement frequency is always expected to break down associations due to an increase of recombination rate. So, the likely explanation would be that fluctuations do not affect associations or at least not in the same way for every studied rearrangement polymorphism.

On the other hand, we favor the general idea that the positive correlation between arrangements and TE copies is not due to an inversion effect but, most probably, to the founder event [19,25,54,55]. This could explain why arrangement E_1+2+9+12_and the 74D site, which is located outside of the inversion, show a positive association and also why an excess of classes including positive correlation coefficients between chromosomal sites was observed in some chromosomes like E. Genetic estimates suggest that the number of founders ranged from 10 to 150 [25,56]. If some founders carried together this site and this arrangement, both will appear together in all populations because they are identical by descent. The founder hypothesis is favored by the fact that all correlations between sites and arrangements are significant only in colonizing populations. In the original population in spite of having correlation coefficients of 0.57 (in 57D) and 0.56 (in 83C) with E_1+2+9 _and O_3+4+7 _respectively, these are not significant. In fact, these two arrangements are currently decreasing in frequency in the Mediterranean populations and perhaps these combinations descend also from a few individuals. All these considerations suggest that most of the associations detected are due to a founder effect.

The general rule, as reported in *D. melanogaster *[45,57], is that TEs are spread and have low insertion frequencies in euchromatin. In some cases, however, accumulations of TEs in some chromosomal sites have been reported, as in the 42B [58], 87C [59] and 38 [60] regions, of *D. melanogaster*, and the 85D region of *D. subobscura *[61,62], and even fixation has occurred, as in the 42C site in natural populations of *D. simulans *[63]. Preferential insertion sites (hotspots) have been suggested for some Drosophila elements [64-66] and we cannot completely discard the possibility of an activation of transposition to specific hotspots during the colonization process. This hypothesis could be verified if a process affecting equally the two TEs studied occurred, as shown in *D. melanogaster*. In this species some proteins are involved in RNA-silencing mechanisms for retrotransposable elements repression [67-69]. We cannot discard the existence of a similar mechanism in *D. subobscura *that was de-repressed as a consequence of the colonization process contributing simultaneously to an increase of transposition of different transposable elements.

### Factors affecting TEs distribution in *D. subobscura*

In Drosophila, TEs seem to be maintained in populations as the result of a balance between transposition and opposing forces that reduce their copy number. In this way selection can act either directly against deleterious insertions or indirectly against deleterious chromosomal rearrangements produced by ectopic recombination between TEs [4,5,36,70]. In this work a test of selection against deleterious insertions was done by comparing copy numbers between X and autosomes, selection being more effective in the former than in the latter.

For *gypsy *we observe a clear tendency to follow a selection model, except in Maipú. This result is in concordance with that observed in a natural population of *D. melanogaster *with this element [71]. For *bilbo *the data do not fit a selection model against deleterious insertions; even in those cases where the test is significant, a higher copy number on A (X), compared to autosomes, is observed. A possible explanation of this result is that *bilbo *could have a differential transposition rate between X and autosomes. Some examples of transposition restricted to female or male *D. melanogaster *germ line have been reported [72,73] and they should be taken into account when X and autosomes are compared. On the other hand, the discrepancies observed between the two elements may be accounted for by the different factors that control copy numbers in each of these elements. In *D. melanogaster gypsy *is a retrovirus [74] submitted probably to a strong selection effect, its transposition depending on the presence of permissive alleles most likely segregating in natural populations. In *D. subobscura *this retrotransposon seems to be non infectious because current available copies have an apparently inactive *env *region [75], but this does not discard the putative presence of alleles that control its transposition. On the other hand *bilbo *is a LINE element and could be submitted to a soft selection pressure due to its DOA transposition mechanism. Most of the copies are probably deleted and its deleterious capability by transposition is diminished. The model of selection against deleterious insertions has been questioned by some authors [28,48] because neither all ETs nor all populations had a lower insertion frequency on X chromosomes compared to autosomes. However in a later work [76], where the authors reanalyze the data including more results from other species, selection against insertions is considered as the major mechanism of TE copy number control. On the other hand, values of selection coefficients against deleterious mutations could not be comparable to mutations associated to TE insertions. Moreover, deleterious effects of TEs can be species specific and populations may also sometimes suffer TE mobilizations that mask selection effects on TE distribution.

In this work each element presents a different behavior probably due to their distinct transposition mechanisms. Moreover we should not forget that elements which are stable in some genome conditions could be unstable in others. Recently mobilized TEs and/or colonization events, in populations, could lead to a differential copy distribution between chromosomes, rendering the selection undetected. This could be the case of Maipú, a new colonizing Argentinian population, which shows a distribution pattern for *gypsy *and *bilbo *quite different from the other colonizing populations. In particular, some high insertion frequency sites are more represented, or even exclusive, in this population. It is possible that Maipú was established through a bottleneck of founder flies from Chile as a consequence of a secondary colonization. In this case, we cannot discard the existence of new transposition events in founders induced by the new environmental conditions encountered as previously proposed by other authors [10,11]. If this colonization occurred recently, as indicated by collecting records, selection has not had enough time to act, explaining the discrepancies in this population when comparing A (X) and autosome copy numbers in Table 3 or when this population is included in heterogeneity tests. In addition if TEs are not at equilibrium, departures from random distribution across chromosomes could reflect the insertion pattern rather than the effect of natural selection.

Another model proposed to explain the TE dynamics is the selection against deleterious arrangements produced by ectopic recombination between TEs. In *D. subobscura *accurate measures of recombination rate are not available and it is not possible to calculate a correlation between TE copies and recombination rates. This species has a rich inversion polymorphism in all chromosomes and recombination is reduced in heterokaryotypes. Under this model we expect accumulation of TEs in inverted segments, and in inversion break points or near them. In some cases arrangements include overlapping inverted fragments, often reaching frequencies higher than the standard arrangements, but in other cases, of low frequency arrangements, TE copy number is too low to allow statistical tests. Also recombination between non-overlapping inversions or inversion complexes may also be prevented [77].

We looked for accumulations of *bilbo *and *gypsy *in breakpoints of inversions but only one high insertion frequency *bilbo *site, 67A, coincides with an inversion breakpoint (E_1+2+9+12_). In another case the 89C high frequency site of *bilbo *is located near the inversion O_8 _and shows a significant correlation with O_3+4+8 _arrangement. This is in concordance with several unsuccessful attempts to localize *in situ *hybridization middle repeated sequences in *D. subobscura *inversions breakpoints [61,78]. These data notwithstanding, we cannot discard that other elements may be responsible of chromosomal inversion induction as reported in other Drosophila species [37,38].

## Conclusion

We conclude that the differential distribution of *bilbo *and *gypsy *between original and colonizing *D. subobscura *populations, is mainly due to a founder effect occurred during the colonization process of this species. We have shown that both founder effect and inversion polymorphism contribute notably to an excess of positive correlations between site pairs. Moreover the two transposable elements show a different pattern of distribution in populations that might be due to their differences in transposition and copy number regulatory mechanisms. This paper is also an attempt to emphasize the importance of population structure and history to explain the TE chromosomal distribution. We highlight the fact that comparisons in TE copy number between X and autosomes have to be interpreted cautiously. Sometimes TEs mobilizations can mask the effect of selection on TE distribution.

## Methods

### Drosophila strains

The control strain *chcu *carries the recessive markers cherry eyes and curled wings and is homokaryotypic for chromosomal arrangements A_st_, J_st_, U_st_, E_st _and O_3+4_. It is kept by mass-culturing to maintain its viability. *In situ *hybridization for insertions of *bilbo *and *gypsy *displayed high stability over generations in 19C, 46A, 46C, 73A, 81D, 84A, 96A for *bilbo *and in 7C and 52A for *gypsy*.

The original population was sampled in Spring 2005 in Bordils (42.30°N, Girona, Spain). The colonizing populations were sampled in Spring 2004 in Davis (38.33°N, California, USA) and Bellingham (48.45°N, Washington, USA), and in Spring 2005 in Maipú (36.52°S, Argentina).

### Mating system (prior to "*in situ*" hybridization)

Individual males of natural populations were crossed with virgin females of the control line *chcu*. Insertion profiles were analyzed in F_1 _female larval progeny to include the X chromosome. The TE insertion profile of each male was deduced by subtracting the TE insertion profile of the control line from that of the F1 larva.

### *In situ *hybridization and DNA probes

Polytene chromosome [16] squashes from salivary glands of third-instar larvae, prepared as described in [79], were hybridized with digoxigenin labelled probes of *bilbo *and *gypsy*. The probes consisted of PCR fragments (2.6 and 2.8 kb long) which included the reverse transcriptase region. Prehybridization solutions and posthybridization washes were done following a protocol by Roche [80]. PCR reactions were carried out in a final volume of 25 μl, including 1× activity buffer (Ecogen), 1.6 mM MgCl_2_, 0.2 mM of each dNTP (Roche), 0.4 μM primer (Roche), 10–20 ng of genomic template DNA, and 0.04 units per μl of Taq polymerase (Ecotaq from Ecogen). Amplifications were run in a MJ Research Inc. thermocycler programmed as follows: 5 min preliminary denaturation at 94°, 30 cycles of 45 s at 94° (denaturation), 45 s at specific PCR annealing temperatures, 1.5 min at 72° (extension) and a final extension for 10 min at 72°. PCR products were gel purified with a Geneclean kit (BIO 101) and labelled using the random primer method. After hybridization signal development was done using an anti-digoxigenin antibody conjugated with alkaline phosphatase (Roche).

*In situ *hybridization is the more suitable method used in localization of TEs on chromosomal arms. However, the power of resolution of this technique allow us neither discriminate between closely neighbouring sites, nor between elements that diverge below 10%.

### Statistical analyses

Statistical analyses were performed excluding centromeric and pericentromeric TEs insertions. The statistical software SPSS version 14.0 was used for most of the statistical data analyses.

In cases of multiple testing, corrections were achieved measuring the significance of False Discovery Rates [81] through q values. To get the q-value we used the software QVALUE [82] on the p values obtained from the multiple test. When this test could not be applied, Bonferroni's correction was performed [83].

## Authors' contributions

MPGG participated in the design, the chromosomal slides, some statistical analyses and the writing of the manuscript. BECS collected the Bordils population, performed most of the technical work, the reading of slides and some statistical analyses. JB collected Davis and Bellingham populations, supervised all arrangement readings and performed the data set analyses. LS collected the Bordils population, contributed to the design and thoroughly revised the manuscript AF participated in the design, directed the project, coordinated the data analyses, contributed to the writing of the manuscript and collected the Maipú and Bordils populations. All authors read and approved the final manuscript

## Supplementary Material

Additional file 1 **Poisson distribution: raw data of bilbo copy number per chromosome and population, P values and chi tests**. A table of detailed tests of Poisson distribution of bilbo per chromosome and haploid genome. Click here for file

Additional file 2 **Poisson distribution: raw data of gypsy copy number per chromosome and population, P values and chi tests**. A table of detailed tests of Poisson distribution of gypsy per chromosome and haploid genome. Click here for file
